# Association of peripheral blood neutrophil gelatinase‐associated lipocalin levels with bone marrow neutrophil gelatinase‐associated lipocalin levels and neutrophil count in hematologic malignancy

**DOI:** 10.1002/jcla.22920

**Published:** 2019-05-15

**Authors:** Chi‐Hyun Cho, Jung Yoon, Deok‐Su Kim, Shin‐Jong Kim, Hwa Jung Sung, Se Ryeon Lee

**Affiliations:** ^1^ Department of Laboratory Medicine, College of Medicine Korea University Seoul Korea; ^2^ Department of Hematology, College of Medicine Korea University Seoul Korea

**Keywords:** bone marrow, hematologic, malignancy, neutrophil, NGAL, peripheral blood

## Abstract

**Background:**

Although neutrophil gelatinase‐associated lipocalin (NGAL) is a biomarker for acute kidney injury, recently, high NGAL levels have been reported in hematologic malignancies. Given the mechanism underlying NGAL synthesis and secretion in neutrophilic series, it is speculated that NGAL levels are higher in bone marrow (BM) than in peripheral blood (PB). Additionally, PB NGAL levels are thought to be associated with neutrophilic parameters. We aimed to test both hypotheses in hematologic malignancies.

**Methods:**

Paired BM and PB samples were collected from 41 patients undergoing BM examination for hematologic malignancies. NGAL levels were measured using immunoassays. Data on hematologic parameters were collected from medical records. Single and multiple regression analyses were performed to analyze the relationship.

**Results:**

PB and BM NGAL (n = 41) levels were significantly different (163.0 ± 258.3 and 413.1 ± 616.2 ng/mL [mean ± standard deviation], respectively; *P* < 0.05). Simple regression analysis and multicollinearity assessment showed that BM NGAL levels, BM neutrophil%, and neutrophil count were significant predictors of PB NGAL. Two multiple regression models were developed (model 1, PB NGAL = 21.467* neutrophil count ‐ 0.785*BM neutrophil%; model 2, PB NGAL = 21.202*neutrophil count‐ 0.915*BM neutrophil% +0.10*BM NGAL). Akaike's information criterion and adjusted *R* ^2^ values showed that model 1 had higher predictive accuracy for PB NGAL. In both models, neutrophil count was the only significant predictor.

**Conclusion:**

BM NGAL was significantly higher than PB NGAL in hematologic malignancy. In addition, PB NGAL could be expressed as a multiple regression model including neutrophil count and BM neutrophil%, being significantly influenced by neutrophil count.

## 1 INTRODUCTION

Neutrophil gelatinase‐associated lipocalin (NGAL, also known as human neutrophil lipocalin, lipocalin 2, siderocalin, uterocalin, proteinase‐3, 24p3, and neu‐related lipocalin), a member of the lipocalin family, is a glycoprotein that was originally purified and characterized from the granules of human neutrophils.^1^, ^2^ NGAL is a biomarker for acute kidney injury; however, recent evidence suggests that NGAL also involved in regulating iron‐responsive genes during cell proliferation and differentiation, and is expressed in other types of tissues in response to various pathologic conditions such as ischemia, tissue injury, and cancer.^1^, ^3^, ^4^ Numerous studies have recently investigated the dysregulation of NGAL in hematologic malignancies.^1^, ^5^, ^6^, ^7^, ^8^, ^9^, ^10^, ^11^, ^12^

Although neutrophils, monocytes/macrophages, and adipocytes display NGAL expression, immature neutrophils, importantly, also have high NGAL expression.^13^ NGAL is normally synthesized as a component of late granules in neutrophils.^1^ NGAL is synthesized during the early stage of neutrophil maturation, but its synthesis stops with the induction of neutrophil maturation.^1^ Differentiated neutrophils have defects in NGAL synthesis and storage. Given the mechanism underlying NGAL synthesis and storage, NGAL levels are thought to be related to hematologic parameters, such as neutrophil count. It is also speculated that NGAL levels are higher in bone marrow (BM) than in plasma, because most neutrophilic precursors are found in the bone marrow. However, to our knowledge, no study has attempted to measure NGAL in both human BM and peripheral blood (PB) or to analyze the relationship between PB NGAL levels and hematologic parameters, including neutrophil count.

Accordingly, to verify these two hypotheses, this study aimed to investigate the difference between BM and PB NGAL levels and analyze the association of PB NGAL levels with hematologic parameters, including neutrophil count, in patients with hematologic malignancies.

## 2 MATERIALS AND METHODS

### 2.1 Sample collection and preparation

The study was conducted according to the Declaration of Helsinki. The experimental protocol was approved by the Institutional Review Board (IRB) of the Korea University Ansan Hospital (No.: AS15066). Patients were enrolled from June to November 2015. Informed consent forms were obtained from every patient who participated in the study (n = 41). Aliquots of leftover BM aspirates and PB samples were collected from 41 patients who underwent BM examination for diagnosis and monitoring of hematologic malignancies at the Korea University Ansan Hospital. Patients were classified into disease groups based on the World Health Organization diagnostic criteria for myeloid proliferative neoplasm (MPN), acute myeloid leukemia (AML), myelodysplastic syndrome (MDS), plasma cell neoplasm (PCN), and lymphoma without BM involvement. Paired PB plasma and BM aspirate supernatants were collected from tubes containing ethylenediaminetetraacetic acid (EDTA) after centrifugation 2399 *g*, 10 minutes and stored at −80°C until analysis. NGAL levels in PB and BM aspirate samples were analyzed simultaneously, using an immunoassay, on an automated platform.

### 2.2 Clinical data collection

Clinical data were collected from electronic medical records for baseline demographics and hematologic parameters such as hemoglobin, white blood cell (WBC) count, neutrophil count, platelet count, BM cellularity, myeloid:erythroid (M:E) ratio, and BM cell% (BM cell counting on BM aspiration slides). BM cell% included BM blast%, BM promyelocyte%, BM myelocyte%, BM metamyelocyte%, BM band neutrophil%, and BM neutrophil%.

### 2.3 NGAL immunoassay

PB plasma samples were analyzed using a mouse monoclonal anti‐NGAL (human) antibody (BioPorto Diagnostics) using a particle‐enhanced turbidimetric immunoassay performed on a Cobas 8000 automation platform (Roche Diagnostics), according to the manufacturer's instructions. The level of NGAL was measured with an automated sequence of 5‐minutes incubation using 3 μL of sample and 150 μL of reaction buffer, followed by another 5‐minutes incubation using 50 μL of immunoparticle suspension. NGAL concentration was calculated from changes in absorbance, based on a calibration curve prepared using calibration results of known concentrations (50‐3000 ng/mL).

### 2.4 NGAL recovery and linearity tests using BM supernatants

According to the manufacturer's instructions, measurements of only plasma and urine samples were validated, while measurement of BM supernatant using the NGAL turbidimetric immunoassay (Roche Diagnostics) needed a validation test, including recovery and linearity tests.

The recovery test was performed as follows: First, NGAL calibrator 5 (BioPorto Diagnostics) with a concentration of 3000 ng/mL was used as a spiking stock solution. Next, three labeled tubes were prepared in two aliquots: “unspiked” (1.0 mL of BM supernatant), “spiked” (0.98 mL of BM supernatant + 20 µL of spiking stock solution), and “control” (0.98 mL normal saline + 20 µL of spiking stock solution).

For the linearity test, serial dilutions of the spiked sample were made. To make 1:2 diluted spiked sample, 0.5 mL of the spiked sample was added to 0.5 mL of saline. Next, 0.5 mL of the 1:2 diluted spiked sample was added to 0.5 mL of saline to make the 1:4 diluted spiked sample. The same procedure was repeated to obtain 1:8 and 1:16 diluted spiked samples. Like the recovery test, two aliquots were prepared from each dilution, and NGAL levels were examined.

%Recovery was calculated as follows:

For the linearity test, %Recovery (1:2), %Recovery (1:4), %Recovery (1:8), and %Recovery (1:16) were calculated as follows:

If %Recovery and linearity fell within the acceptance range of 80%‐120%, the feasibility of using the NGAL turbidimetric immunoassay (BioPorto Diagnostics) for BM supernatant would be validated.

### 2.5 Statistical analyses

The paired t test and Wilcoxon's signed rank test were used to compare NGAL levels in PB and BM. A simple regression analysis was performed to analyze the relationship of PB NGAL levels with each hematologic parameter, including BM NGAL levels. A multiple regression analysis was then performed to analyze the relationship of PB NGAL levels with all the hematologic parameters simultaneously, while multicollinearity was analyzed to identify closely related independent variables. When independent variables presented variance influence factor (VIF) values >10, those independent variables were considered to have multicollinearity. The predictive accuracy of the multiple regression models was assessed using Akaike's information criterion (AIC) and adjusted *R* ^2^ values.^14^, ^15^ Statistical significance was set at *P* < 0.05. Wilcoxon's signed rank test was carried out with *P* values at <0.01 (=0.05/5). SPSS version 21.0 (SPSS) was used for all statistical analyses.

## 3 RESULTS

### 3.1 Patient characteristics

Paired BM and plasma samples were collected from 41 patients with a median age of 63 (range 35‐88) years; 56.1% (23/41) of the patients were men, and 43.9% (18/41) were women (Table 1). The underlying diagnoses were MPN (n = 12), AML (n = 5), MDS (n = 13), PCN (n = 6), and lymphoma without BM involvement (n = 5). Samples (n = 12) of MPN consisted of CML (n = 4), polycythemia vera (PV) (n = 7), and essential thrombocythemia (ET) (n = 1). Of the 41 patients, 40 were at the initial diagnosis stage. One patient was previously diagnosed with MDS with fibrosis, but only received supportive treatment and was rediagnosed with refractory anemia with excess blasts‐2 and fibrosis in a follow‐up BM study. The patients’ hematologic parameters are presented in Table 1.

**Table 1:** Patients’ demographic features and laboratory parameters

Characteristics	Value
Age, (y) (median [range])	63 (35‐88)
Male/female	23/18
Hb (g/L)	101 (54‐213)
WBC count (median [range]) (10^9^/L)	5.76 (0.79‐119.96)
Neutrophil counts (median [range]) (10^9^/L)	3.34 (0.11‐70.78)
Platelet count (10^9^/L)	175 (18‐1937)
BM cellularity (median [range])	55 (10‐98)
M:E ratio (median [range])	2.5 (0.3‐76)
Disease entities (n = 41)	MPN^a^ (n = 12)
	AML (n = 5)
	MDS (n = 13)
	PCN (n = 6)
	Lymphoma^b^ (n = 5)

Abbreviations: AML, acute myeloid leukemia; MDS, myelodysplastic syndrome; MPN, myeloproliferative neoplasm; PCN, plasma cell neoplasm.

a MPN included CML (n = 4), ET (n = 1), and PV (n = 7).

b lymphoma without bone marrow involvement.

### 3.2 NGAL recovery and linearity tests using BM supernatants

For the recovery and linearity tests, two BM supernatant samples were used. For the first sample, %Recovery was 98.25%, and linearity results showed that %Recovery (1:2), %Recovery (1:4), %Recovery (1:8), and %Recovery (1:16) were 99.6%, 103.96%, 108.44%, and 96.28%, respectively.

For the second sample, %Recovery was 85.08%, and linearity results showed that %Recovery (1:2), %Recovery (1:4), %Recovery (1:8), and %Recovery (1:16) were 103.06%, 105.71%, 110.12%, and 109.06%, respectively. For both BM supernatant samples, both recovery and linearity test results fell within acceptable range, validating the feasibility of using NGAL turbidimetric immunoassays (BioPorto Diagnostics) for the analysis of these samples.

### 3.3 PB and BM NGAL levels, examined using immunoassays

The overall PB and BM NGAL concentrations (n = 41) were 163.0 ± 258.3 and 413.1 ± 616.2 ng/mL (mean ± standard deviation [SD]), respectively. NGAL levels in paired BM and PB samples were significantly different. In the MPN group, BM NGAL levels (mean ± SD, 1184.9 ± 1045.0 ng/mL) were significantly higher than PB NGAL levels (329.0 ± 417.1 ng/mL) (*P* = 0.001).

### 3.4 Simple regression analysis of the relationship of PB NGAL levels with BM NGAL levels and hematologic parameters

Simple regression analysis identified four significant predictors of PB NGAL levels: BM NGAL levels, BM neutrophil%, neutrophil count, and WBC count (Table 2). Next, the multiple regression analysis showed multicollinearity in WBC (VIF = 28.801) and neutrophil counts (VIF = 29.271). WBC count was removed from the predictor variables, as PB NGAL levels were considered to have a stronger association with neutrophil count than with WBC count, according to mechanism underlying NGAL synthesis.

**Table 2:** Simple regression analysis of peripheral blood neutrophil gelatinase‐associated lipocalin levels with hematologic parameters in hematologic cancers

Clinical parameters	*R* ^2^	*P‐*value
Age	0.070	0.095
Hb (g/L)	0.008	0.583
WBC (10^9^/L)	0.917	0.000*
Neutrophil (10^9^/L)	0.905	0.000*
Platelet count (10^9^/L)	0.021	0.365
M:E ratio	0.002	0.790
BM blast%	0.031	0.270
BM promyelocyte%	0.003	0.744
BM myelocyte%	0.047	0.174
BM metamyelocyte%	0.018	0.408
BM band neutrophil%	0.075	0.083
BM neutrophil%	0.184	0.005*
BM cellularity	0.041	0.205
BM NGAL (ng/mL)	0.242	0.001*

Abbreviations: BM, bone marrow; Hb, hemoglobin; M:E ratio, myeloid precursors:erythroid precursors ratio; PB, peripheral blood.

* Statistically significant (*P* < 0.05).

The multiple regression analysis used the three remaining independent variables **(**BM NGAL levels, BM neutrophil%, and neutrophil count), and two multiple regression models were developed as follows (Table 3).

**Table 3:** Regression analysis models of the relationship of peripheral blood (PB) neutrophil gelatinase‐associated lipocalin (NGAL) levels with bone marrow (BM) NGAL levels and hematologic parameters in hematologic malignancies

	Coefficient	*t*‐value	*P*‐value	Adjusted *R* ^2^	AIC
Model 1					
Neutrophil count	21.467	17.039	0.000*	0.901	362.577
BM neutrophil%	−0.785	−0.570	0.572		
Constant	33.181	1.358	0.182		
Model 2					
Neutrophil count	21.202	15.324	0.000*	0.899	364.312
BM neutrophil%	−0.915	−0.646	0.523		
BM NGAL levels	0.10	0.489	0.628		
Constant	32.432	1.311	0.198		

Abbreviations: Adj, adjusted; AIC, Akaike's information criterion.

* Statistically significant (*P* < 0.05).

(Model 1) PB NGAL = 21.467* neutrophil count ‐ 0.785*BM neutrophil%.

(Model 2) PB NGAL = 21.202*neutrophil count‐ 0.915*BM neutrophil% +0.10*BM NGAL.

For each model, adjusted *R* ^2^ and AIC values are presented in Table 3. The determined AIC value was lower for model 1 than for model 2, and the adjusted *R* ^2^ value for model 1 was higher than that for model 2. This suggests that model 1 had a higher predictive accuracy for PB NGAL levels than did model 2 (Table 3).^14^, ^15^ In both models, the only significant independent variable was neutrophil count (*P* = 0.000).

## 4 DISCUSSION

In this study, two hypotheses based on the mechanism underlying NGAL synthesis were verified using samples from patients with hematologic malignancies. The first hypothesis was that PB NGAL levels are higher than BM NGAL levels, and the second was that PB NGAL levels are associated with neutrophil count.

To carry out the NGAL immunoassay (BioPorto Diagnostics), a validation test was required for the BM supernatant sample, as manufacturer's instructions mention that the reagent can only be used for plasma and urine samples. This study was the first attempted validation test for this reagent. Since the recovery and linearity test results were acceptable, the BM supernatant sample could be examined using this reagent.

The comparison of paired BM and PB NGAL levels showed that BM NGAL levels were higher than PB NGAL levels. As sources of NGAL, neutrophilic precursors (such as promyelocytes, myelocytes, and metamyelocytes) are mainly found in the BM, but not in the PB. Accordingly, some studies reported that the metamyelocyte‐enriched BM fraction exhibits strong NGAL expression.^1^ This might explain why the levels of BM NGAL were higher than those of PB NGAL.

BM NGAL levels were much higher than PB NGAL levels in the MPN group. In MPN, myeloid precursors proliferate mainly in the BM, and to a much lower extent in PB. Therefore, the difference between BM and PB NGAL levels was more prominent in the MPN group than in total.

The simple regression analysis identified BM NGAL levels, BM neutrophil%, neutrophil counts, and WBC counts as independent predictors of PB NGAL levels (Table 2). Among them, WBC (VIF = 28.801) and neutrophil (VIF = 29.271) counts showed multicollinearity. As NGAL is mostly synthesized and only stored in neutrophilic precursors, and is secreted in mature neutrophils, logically, one would deduce that the factor affecting PB NGAL levels would be neutrophil count and not WBC count.^13^ Accordingly, WBC was removed from the list of independent variables, and the multiple regression analysis was performed using only BM NGAL levels, BM neutrophil%, and neutrophil count as independent variables.

Nevertheless, the question remains whether BM NGAL levels should be included as an independent variable in the multiple regression analysis. This is because the peripheral blood system where PB NGAL is present is connected through sinusoids to the bone marrow system, where BM NGAL is present. Furthermore, BM aspirates may be diluted with PB during BM aspiration and biopsy procedures. Therefore, we cannot exclude the possibility that PB NGAL and BM NGAL might be inter‐related entities existing in connected spaces. To address this limitation, we developed two multiple regression analysis models: one with BM NGAL levels as an independent variable (model 2, Table 3) and the other without (model 1, Table 3).

The multiple regression analysis showed that model 1 had higher adjusted *R* ^2 ^values and lower AIC values than model 2, and thus had a higher predictive accuracy for PB NGAL levels than did model 2 (Table 3). Additionally, model 1 was more compatible with the mechanism underlying NGAL synthesis, as PB NGAL synthesis is associated with absolute neutrophil count and BM neutrophil%.

PB NGAL levels were not significantly related to BM blast%, BM promyelocyte%, BM myelocyte%, BM metamyelocyte%, or BM band neutrophil% (Table 2). This reflects the fact that NGAL is synthesized and stored mainly in neutrophilic precursors, but is secreted mainly from mature neutrophils.^1^ In other words, PB NGAL levels are not associated with BM neutrophilic precursor%, but with mature neutrophils or BM neutrophil%. Additionally, neutrophil count was the only significant predictor in both multiple regression models, suggesting that PB NGAL levels could be affected by neutrophil count.

In conclusion, using samples from patients with hematologic malignancies, we found that BM NGAL levels were significantly higher than PB NGAL levels, supporting the fact that NGAL is synthesized in neutrophilic precursors, which are present mainly in BM. Additionally, PB NGAL levels can be predicted by absolute neutrophil count and BM neutrophil%, while neutrophil count is the only significant independent variable. When PB NGAL levels are measured in clinical conditions, neutrophil count should be considered as an important influencing factor.
